# The influences of group dialog on individual student understanding of science concepts

**DOI:** 10.1186/s40594-018-0142-3

**Published:** 2018-11-03

**Authors:** Abdi-Rizak M. Warfa, James Nyachwaya, Gillian Roehrig

**Affiliations:** 1 Department of Biology Teaching and Learning, University of Minnesota, Minneapolis, MN 55455 USA; 2 Department of Chemistry and Biochemistry & School of Education, North Dakota State University, Fargo, ND 58108 USA; 3 STEM Education Center, Department of Curriculum and Instruction, University of Minnesota, St Paul, MN 55108 USA

**Keywords:** Discourse analysis, Sociochemical norms, Cooperative learning, College chemistry, Chemistry education

## Abstract

**Background:**

Cooperative and inquiry-based pedagogies provide a context for classroom discourse in which students develop joint understanding of subject matter knowledge. Using the symbolic interactionist perspective that meaning is constructed as individuals interact with one another, we examined how student groups enrolled in an undergraduate general chemistry course developed sociochemical norms that influenced individual student understanding of chemical concepts. Sociochemical norms refer to the normative aspects of classroom microculture that regulate discourse on what counts as a table chemical justification and explanation. We describe how these sociochemical normative ideas were developed based on observational research and recordings of the student groups as they engaged in classroom discourse.

**Results:**

Our analysis showed that students routinely developed chemistry-driven criteria within and across groups to explain the nature of dissolving ionic solids in water. Moreover, resultant sociochemical norms led to shifts in student understanding and the ways in which students reasoned about the causes of chemical phenomena under study.

**Conclusions:**

Our results indicate that group dialog influenced individual student conceptions of ionic compounds in solution and highlight the need to engage students in instructional activities that not only engage them in the multiple ways of representing chemical knowledge but also making public their views and participating in classroom discourse.

**Electronic supplementary material:**

The online version of this article (10.1186/s40594-018-0142-3) contains supplementary material, which is available to authorized users.

## Introduction

For more than two decades, there has been a gradual paradigm shift towards cooperative and inquiry-based pedagogies in college science teaching (Paulson 1999; Bowen 2000; Spencer 2006; Eilks and Byers 2009; Warfa 2015). The success of these pedagogies in the teaching of science in part stem from their ability to foster student discussion and create collaborative learning dynamics in a social context wherein problem-solving and conceptual understanding is stressed (Johnson et al. 1991). For example, POGIL® (process oriented guided inquiry learning) is a pedagogical strategy that promotes students working collaboratively on guided materials that emphasize understanding core chemical concepts but also developing higher-order thinking skills (Farrell et al. 1999; Spencer and Moog 2008). Analyses of student achievement and learning gains have shown both cooperative learning and the POGIL approach having significantly a positive impact on student learning in chemistry (Paulson 1999; Lewis and Lewis 2005; Bowen 2000; Warfa 2015; Walker and Warfa 2017). Consequently, cooperative learning and inquiry approaches have been widely embraced by the chemical education community.

One-way cooperative and inquiry-based pedagogies enhance student learning are the opportunities they offer to enact classroom discourse practices that allow students to develop joint understanding of core chemical ideas (Paulson 1999; Eilks and Byers 2009; Becker et al. 2013). For example, the structure of POGIL activities requires students to play specific roles within their group (i.e., facilitator, recorder, skeptic, etc.) and elicits group construction of concept understanding and knowledge growth. The benefits of such collaborative discourses in science learning is well documented (Osborne 2010). However, it is less clear how such social interactions and group thinking influence individual student understanding.

In the present study, we used a POGIL activity designed to target student misconceptions related to chemical bonding to examine how group thinking during cooperative activities influenced college students’ conceptions of ionic compounds in solution. The structure of the activity was such that students were in situated events that encouraged them to verbalize their thinking, discuss particulate and symbolic representations of ionic and molecular compounds, and make sense of chemical ideas. In the paper, we analyze how these social interactions influenced how groups constructed the meaning of chemical bonding and the ways in which students developed individual understandings. The guiding research question for this study was: *What sociochemical norms do student groups develop to describe the dissolution of ionic compounds in water*?

To answer our research question, we used the construct of sociochemical norms (Becker et al. 2013) to first identify patterns of reasoning and explanations within and across ten different autonomous cooperative learning groups in a general chemistry course (*N* = 84). Subsequently, we examined whether unfolding sociochemical dialogs in the groups led to shifts in student understanding with respect to the properties of dissolved ionic solids in water. As we describe below, several authors (Ebenezer and Erickson 1996; Kelly and Jones 2007; Tien et al. 2007; Naah and Sanger 2012, 2013) have examined students’ understanding of the dissolution process of ionic compounds and cataloged several student misconceptions. Our paper contributes to this literature in that it analyses how classroom discourse within a POGIL activity influences individual student’s conceptions of the dissolution process and provides a methodological approach to study the influences of classroom discourse on student science learning rather than simply cataloging student misconceptions.

## Background literature

### Sociochemical norms

Becker et al. (2013) coined the term *sociochemical norms* to describe the disciplinary criteria that regulate classroom discourse and normative aspects in the study of chemistry. This is an extension of the social construct of *sociomathematical norms* (Yackel and Cobb 1996) proposed as a way to interpret how mathematics classroom dynamics affect student development of mathematical beliefs and values. In contrast to general classroom social norms, sociomathematical norms refer to “normative aspects of mathematics discussions specific to students’ mathematical ability” (Yackel and Cobb 1996, p. 461). While the distinction between social and sociomathematical norms is subtle, it is clear the latter is a disciplinary criterion for *what counts* as an acceptable mathematical explanation and justification whereas social norms refer to expected general classroom customs, e.g., the ability to explain a problem or ways of thinking about the problem (Yackel and Cobb 1996).

Given the disciplinary nature of sociomathematical norms, Cole et al. (2012) studied classroom discourse in an undergraduate physical chemistry course. The authors used the term *classroom chemistry practice* to refer to the normative ways of reasoning that develop as students work together to solve problems, explain their thinking, and entertain opposing points of view. For instance, particulate-level descriptions of solids, liquids, and gases became “central to the collective reasoning about thermodynamic concepts and processes” (Cole et al. 2012, p.206). This particulate-level reasoning forms discipline-specific justification for what counts as chemically justifiable and acceptable and hence a normative aspect of classroom discourse specific to the study of chemistry. In their subsequent paper, Becker et al. (2013) used the term *sociochemical norms* to describe these disciplinary-specific normative ways of reasoning. In this paper, we use this construct to examine how students negotiate what counts as an acceptable chemical explanation, reasoning, and justification for the physical and chemical properties of dissolved ionic solids.

In addition to the concept of sociochemical norms, our group recently used the term *sociochemical dialogs* to better characterize the dialogs regulated by class-established sociochemical norms as envisioned by Becker and colleagues (Warfa et al. 2014). In our view, sociochemical dialogs occur in chemistry classrooms that are often regulated by discipline-specific norms and discourse. Thus, in this paper, we refer to the dialogs occurring in the classroom we observed as sociochemical dialogs.

### Representational fluency

One difficulty in understanding chemical bonding is related to the multiple levels of representations used in chemistry to describe and explain chemical bonding (Gabel 1998; Johnstone 1991). Chemical bonds can be represented by using particulate drawings, abstract symbols or chemical formulas, or through macroscopic properties (Johnstone 1991). These representational forms are not exclusive to each other, but rather interconnected (Harrison and Treagust 2002; Naah and Sanger 2012, 2013). The challenge, however, has been students’ inability to shift between these different representations and develop conceptual understanding. Given how prevalent symbolic representations are in the teaching of chemistry, we argue this appears to be a question of pedagogical approach. To promote students’ conceptual understanding and competency with chemical representations, students need to be in situations that force them to verbalize their ideas (Stinessen 1985; Berardi-Coletta et al. 1995) and construct robust meaning of chemical bonds that incorporates an understanding of bonding at different representational levels (Airey and Linder 2009).

Beyond verbalization of content in representations, the concept of representational fluency contains several aspects that are important for meaningful learning to occur. Airey and Linder (2009) particularly characterize what they call “disciplinary discourse” as the complex of representations, tools, and activities of discipline that are necessary for university science learning. According to Airey and Linder (2009), disciplinary discourse is made up of various modes such as spoken and written language, mathematics, gesture, images (e.g., pictorial representations), tools, and activities (e.g., ways of working). Fluency in these different modes of disciplinary discourse is a necessary condition for developing meaningful learning of science concepts.

### Student conceptions of ionic compounds in solution

Several authors have studied the dissolution of ionic compounds and revealed various student misconceptions, including the notion that *dissolved ionic compounds react with water to form an acid and a metal* oxide (Kelly and Jones 2007; Naah and Sanger 2012; Tien et al. 2007), *ionic solids dissolve as neutral atoms or molecules* (Ebenezer, 2001; Naah and Sanger 2012; Nyachwaya et al. 2011; Tien et al. 2007), *polyatomic ions dissociate as discrete ions* (Naah and Sanger 2012; Nyachwaya et al. 2011; Tien et al. 2007), and a general *confusion about oxidation states, subscripts, and coefficients in chemical equations* (Naah and Sanger 2012; Nyachwaya et al. 2011; Tien et al. 2007). For this study, we were interested in understanding how group sociochemical dialogs shifted students’ thinking and conceptions of the dissolution ionic solids from these documented misconceptions.

## Theoretical frameworks

The guiding theoretical framework for this study considers two distinct perspectives on knowledge acquisition: the symbolic interactionist perspective advanced by Blumer (1969) and diSessa’s (1988, 1993) knowledge in pieces framework. In the symbolic interactionist framework, individuals are thought to construct meaning through interaction with one another by sharing individual perspectives or developing common definitions through group negotiation (Bogdan and Biklen 2003). There are two reasons why we find symbolic interactionism useful for our purposes. First, our research is conducted in a cooperative inquiry-oriented setting in which face-to-face interactions and positive interdependence are essential features of the classroom micro-culture (Johnson et al. 1991; Spencer and Moog 2008). Furthermore, symbolic interactionism allows for the examination of “how individuals are able to take one another’s perspective and learn meanings and symbols in concrete instances of setting” (Jacob 1987, p.29). diSessa’s “knowledge in pieces” framework considers knowledge to consist small elements or “resources” that activate or do not activate, depending on context. The perspective can explain why students may hold different or alternate conceptions about a chemical phenomenon depending on which knowledge pieces are activated and would align better to the productive dialog we see in the multiple groups we observed. It is particularly relevant to the context of our study as the discourse the participants in our study engage may activate relevant resources that enable them to develop appropriate chemical justifications for the dissolution of chemical compounds in water.

## Methods

### Study setting

The data for this study came from an undergraduate general chemistry course taught by a POGIL-trained instructor at a comprehensive regional university in the USA. There were 84 students enrolled in the course, with roughly an equal percentage of males and females. Over half of the instructor’s lecture and discussion sessions centered on small group learning activities. Using the POGIL activity described below, students formed small teams of 3–4 members with self-assigned cooperative roles (i.e., facilitator, recorder, skeptic, etc.). The first author observed all lectures over the course of a 15-week semester, and recordings were done as described below.

### Unit of instruction

**Fig. 1:**
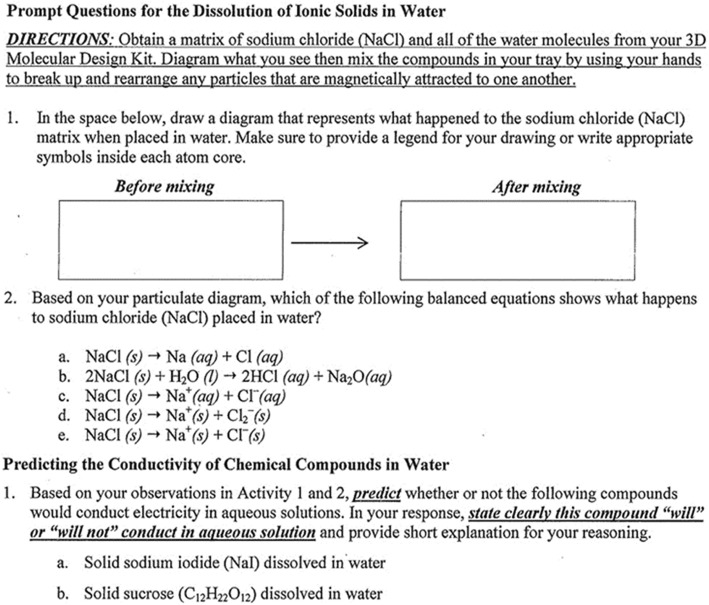
Prompt questions from POGIL ChemActivity used to explore sociochemical norms

Data was collected during a POGIL ChemActivity on chemical bonding. This activity was approved by the POGIL Project (www.pogil.org) and designed to promote representational fluency and to target student misconceptions on the dissolution of ionic compounds described in the “Background literature” section and to promote representational fluency. The activity was divided into four subparts: (1) macroscopic observation of dissolving ionic and molecular compounds in water, (2) tactile experience with model kits (www.3dmolecuardesigns.com) representing the dissolution of ionic salts, (3) tactile experience with model kits representing dissolution of a molecular compound in water, and (4) interpretation of conductivity data of aqueous solutions. Figure 1 shows the prompt questions used in this study. These questions come from subparts 2 and 3 of the activity. We note that the directions given to the students in Fig. 1 instructed them “to break up and re-arrange any particles that are magnetically attracted to one another.” This is, however, an artifact of the model kit that can introduce misconceptions and the course instructor reminded students verbally that ionic forces are Columbic and that all models, including the kit they are using, have certain limitations.

Students spent three class sessions to complete the activity. During the first day, the instructor conducted macroscopic demonstrations of what happens to different chemicals placed in water and led whole class discussions surrounding the demonstration. Subsequently, students used a 3D molecular kit to complete the rest of the activity.

### Data collection

The primary data source was audio-recorded group dialogs in response to the prompts described in Fig. 1. Thirteen of twenty-four student groups, each with 3–4 students, consented to be audio-recorded during the POGIL ChemActivity on chemical bonding. Secondary data included classroom artifacts (e.g., worksheet) and researcher field notes. Due to technical problems, audio files from three of the thirteen recordings could not be used. Thus, the final sample consisted of recordings from ten different autonomous groups. Group and class comparisons during data analysis showed the groups were representative of the whole class (e.g., comparable mean pre-posttest scores for recorded vs. non-recorded participants). All recordings were completed during three separate 1-hour discussion sessions led by the course instructor; the numbering in the groups’ names refers to which session the group attended (session 1, 2, or 3 while the letters A, B, C, and D refer to the groups within each session (e.g., group 2D means group D in session 2).

### Data analysis

Given the dialogical nature of group discussions in POGIL activities, our analysis relied on sequential groups of statements, referred to as episodes, from transcribed group conversations. The use of episodes to understand classroom interactions has a long precedence in educational research (Hollabaugh 1995). Each episode captures the discourse between individuals talking to each other, and therefore, all episodes show individual student turns as part of their sociochemical dialogs. Therefore, the unit of analysis in this study was at the turn level although we parsed the data into episodes. For the purposes of this study, episode boundaries were determined by shifts in what was discussed—such as the initiation of a new topic or new aspects of the same topic (e.g., a shift from a discussion of ionic compounds to molecular compounds). Using these episodes, we looked for specific practices within and across the groups to identify sociochemical norms indicative of group influences on individual understanding. We considered an idea to have become normative if the ten groups used it consistently as a chemical justification. Further refinement of these guidelines led to the coding scheme and criteria shown in Table 1.

**Table 1:** Coding scheme and criteria for data analysis

Code	Code abbreviation	Criteria	Explanation
Group-Negotiated Criteria	GNC	Student groups negotiate and develop a criterion for what counts as acceptable justification for a chemical phenomenon (e.g., the dissolution of ionic compounds)	Code for group constitution of meaning
Across Group-Negotiated Criteria	AGC	• Different autonomous groups develop and use similar criteria to explain the same chemical phenomenon • Same criterion is used as justification for different chemical topics by the same or different groups	Code for a way of thinking becoming normative in class culture

There were two codes, Group-Negotiated Criteria (GNC), 2) and Across Group-Negotiated Criteria (AGC), that we used to code the data (Table 1). The first code, GNC, was used to code statements indicating group construction of meaning. That is, this code was used when we observed student groups negotiating and developing criteria for what counted as acceptable justification for a given chemical phenomenon, such as the dissolution of ionic compounds in water. The second code, AGC, was used for statements that indicated a particular thinking which became normative in small group or whole class discussions. Statements coded as AGC needed to meet one of two criteria: (1) different autonomous groups developed and used similar criteria to explain the same chemical phenomenon and/or (2) different groups used the same criterion as justification for different topics. The criteria for the code AGC was based on modifications of criteria previously developed by Cole et al. (2012) and Becker et al. (2013) and allowed us to compare the criterion the different groups developed and whether, when looking across groups, the participants were developing similar or different justifications.

The coding scheme was collectively revised and fine-tuned during research meetings. Upon agreement on the coding scheme, two researchers coded together an episode from one of the groups (group 1B) and proceeded to code a second portion individually. These researchers then compared their results to establish consistency of code use. Inter-rater reliability based on percent agreement was at 91.7%. Following this initial coding event, other members of the research team coded portions of groups 1B and 2A episodes. Inter-rater reliability was again established, with greater than 90% agreement. Subsequently, one researcher coded the rest of the data, with ongoing discussion with other research members.

## Results

Our results elucidate the specific ways of thinking unique to the study of chemistry that became normative during group dialogs. The following section describes the development of these sociochemical normative ideas and how they were used within and across groups to describe the dissolution processes of chemical compounds. In the interest of space, in each section, we provide illustrative cases followed by a summary across all ten groups.

## Developing own criteria for dissolving ionic compounds

Each group collectively developed criteria for what counts as an acceptable justification for the dissolution of ionic solids in water. The following illustrates cases (group 1A and group 2D) in which groups independently developed similar criteria for selecting the correct balanced chemical equation as they completed the POGIL ChemActivity (see Fig. 1).

### Group 1A’s sociochemical dialog

Group 1A was a homogenous group of four females each playing a different cooperative role. Student 1 (*S1*) was the group’s facilitator, *S2* the spokesperson, *S3* the recorder, and *S4* a process analyst. The group was animated and appeared to have good rapport. Once they took up their different roles, they immediately started discussing the problems in the activity. Table 2 shows the episode in which the group discussed the dissolution process of NaCl in water as well as the prompt question that elicited the group’s discussion.

**Table 2:** Group 1A’s dialog on choosing correct equation for the dissolution of NaCl in water

Dialog	Code	Comments/interpretations
1 SI: So the water didn’t change [points to the molecular model of water in their tray], so the water goes with ... so it’s not A. Are they [pointing to Na and Cl in their drawing] aqueous?	GNC AGC*	Initiating move; SI proposes justification (water didn’t change) and asks for clarification (are they aqueous)
2 S2: Yeah, they’re aqueous	GNC/AGC	Confirmatory response statement
3 S3: So, wouldn’t it be C? Those are the only aqueous	GNC/AGC	Consensus checking statement-justifies why
4 S4: Yeah. That’s what I’d think	GNC	Acknowledgement statement
5 S2: They’re still ions	GNC AGC	S2 proposes a new criteria (needs to be ions)
6 S1: So a plus and negative would be good. And it’s balanced. Good. So now activity three, [end of episode]	GNC/AGC/SST	This is coded both GNC and SST as SI did not initially propose +/-, student shifts thinking in LN 6

In the initiating statement of this episode, *S1* observed that water did not change in the model kit the students used to simulate the dissolution of NaCl in water stating “So, the water didn’t change” (LN 1) and posed to the group a clarification question—“are they aqueous?” (LN 1, Table 2). In response to *S1*’s question, *S2* provided a confirmatory response of “Yeah, they’re aqueous” (LN 2). There was no follow-up on *S1*’s idea that *water did not change* and this criterion was dropped from the further ensuing group discussion. We see in line 3 that *S3* proposed option C of the multiple-choice question as the most likely valid option but sought group consensus as indicated by her statement of “So, wouldn’t it be C? Those are the only aqueous” (LN 3). In proposing option C as the most likely answer, *S3* used the previously discussed idea of “aqueous” as justification for why she thought C was the correct response.

Responding to *S3*, another member of the group, *S4*, acknowledged that C is likely the correct response—“Yeah. That’s what I’d think” (LN 4). In addition to the criterion of dissolving salts being aqueous, the idea of ions as criterion for selecting the correct response is proposed by *S2* when she states “They’re still ions” (LN 5). Following this back and forth dialog, the group used the chemical ideas of “aqueous” and “ions” as a justification for why they selected option C of the multiple-choice question. This is summarized by *S1*, the group’s facilitator, in line 6 of Table 2 as “So a plus and negative would be good. And it’s balanced.”

A key claim in this research is that groups negotiate criteria for what counts as a chemical justification for their explanations. Group 1A’s dialog is characterized by several statement types that support this idea of socially negotiated chemical meaning-making. These include clarification statements posed to the group (*S1*, LN 1), confirmatory response statements (*S2*, LN 2), consensus checking statements (*S3*, LN 3), and acknowledgement statements (*S4*, LN 4). The nature of these discursive practices suggests group 1A was involved in the collective development of chemical justifications to explain the dissolution of NaCl in water.

The chemical ideas of “aqueous” and “ions” as criteria for selecting the correct equation to represent the dissolution of ionic solids in water was not discussed by the course instructor at the beginning of the activity. Rather, this was student-negotiated criteria during the activity. The fact that the group repeatedly used these criteria in their discourse to justify their reason for selecting a particular chemical equation to represent the dissolution process suggests these ideas became normative within the group.

### Group 2D’s sociochemical dialog

The second illustrative example comes from group 2D. This group was homogenous in terms of gender make-up with four males (*S1*–*S4*). *S1* was the group’s facilitator, *S2* the spokesperson, *S3* the recorder, and *S4* the process analyst. The group’s dialog showed animated discussion with members questioning each other’s ideas. Table 3 shows the group’s sociochemical dialog as they justified their reasoning for which multiple-choice option best represented the dissolution of NaCl.

**Table 3:** Group 2D’s dialog on choosing correct equation for NaCl dissolved in water

Dialog	Code	Comments/interpretations
1 S2: Well, it’d have to be mixed because it’s mixed with water, but that doesn’t make sense [points to B as answer]	GNC	Initiating move; proposes an explanation but suggests option B doesn’t make sense
2 S3: I think it’s C because the Na and Cl	GNC/AGC	Follow-up response statement
3 S4: Is it B or D?	GNC/SST	Information request
4 S3: It’s not B	GNC	Response statement
5 S2: It can’t be C because there is no H2O	GNC/AGC	Rebuttal statement on why option C is not correct
6 S3: Does there have to be?	GNC	Skeptical questioning - challenges S2’s claim
7 S4: This one [points to B] has the 2 Na with the 0 because the 0 stays with it	GNC/AGC/SST	Provides explanation of why he thinks B is correct
8 S3: I think it’s just trying to say what happens	GNC	Explanatory statement
9 S4: So you think it’s C?	GNC/SST	Self-doubt after challenged -> SST
10 S3: Because 2 NaCl makes hydrochloric acid and we don’t have that. This one [points to C] shows the charge and makes more sense. If not B then I think that	GNC/AGC/SST	Explanatory statement of why C is correct but self-doubts
11 S3: So, you think it’s C? [Instructor interference]	GNC/SST	Consensus checking statement

In the initiating statement of the episode, *S2* argued that NaCl must be mixed with water although option B did not make sense to him (LN 1). It was not apparent however from the dialog why he thought option B did not make sense. *S3* then puts forward C as the correct option because it showed “Na and Cl” (LN 2). *S4*, another member of the group, asked if the correct response option should be “B or D” (LN 3). *S3* simply stated “it can’t be B” (LN 4) but did not rule out option D. *S2*, on the other hand, refuted *S3*’s suggestion of option C on the basis that “there is no H_2_O” (LN 5). *S2* seemed conflicted: in the initiating statement (LN 1), he argued option B did not make sense but also ruled out option C because “there is no H_2_O” (LN 5).

From the unfolding sociochemical dialog, it is apparent the students in group 2D were thinking about chemical justification for their responses. In the initiating statement, *S2* described the dissolution of ionic salts as “it’d have to be mixed because it’s mixed with water” (LN 1). The correct chemical terminology would have been it ‘became aqueous.’ One can infer from this finding that *S2* probably did not have a good grasp of the nature of aqueous solutions and hence his difficulty in describing the dissolution process. *S4*, on the other hand, interpreted the hydration of sodium ions in the physical model the students used to mean a new bond forming between Na and the oxygen of H_2_O (“This one [*points to B*] has the 2 Na with the O because the O stays with it” LN 7). When *S4* says “this one,” he is using the kit and physically manipulating the NaCl model surrounded by water molecules which led him to think that option B, which shows the addition of water to NaCl, was probably the correct option.

This group appears to use an extensive array of chemical arguments. For instance, when discussing why option B is incorrect, *S3* argued option B does not make sense “because [it says] NaCl makes hydrochloric acid and we don’t have that” (LN 10) and moved on to suggest option C was correct because it “shows the charge and makes more sense” (LN 10). Here, *S3* made a chemical argument against option B (a chemical change would have resulted in the formation of HCl) and an argument for option C (it shows charges). Moreover, all students in the group were involved in the ensuing sociochemical dialog and attempted to convince each other based on chemical reasoning and justification. Thus¸ the students were involved in developing what criteria counts as chemically justifiable for symbolically representing the dissolution of ionic salts in water.

### Extension to remaining eight groups

The preceding cases illustrated how individual groups repeatedly used the ideas of ionic solids in water separating into ions and becoming aqueous as justification for their response. We extended this analysis to the remaining eight groups and found similar ideas repeatedly used as justification by the different groups. Table 4 shows four different criteria developed by the different student groups in this study as a guide for selecting the appropriately balanced chemical equation for dissolved ionic solids in water. As can be seen in the table, the chemical ideas ions, aqueous, chemical change, and the presence of water became normative in the groups’ discourses and were used repeatedly by the majority of the groups. This met one of the criteria set forth to answer our research question: student groups negotiated and developed criteria for what counted as acceptable justification for a given chemical phenomenon. It was also apparent from the groups’ sociochemical dialogs that they were involved in chemical meaning-making and negotiated about what criteria to use for selecting the correct multiple-choice option. That is, their responses were guided by chemical justification and the use of chemical ideas.

**Table 4:** Comparison of group uses of student-generated criteria for the dissolution of ionic solids in water (‘+’ *means group used indicated criterion whereas ‘-’ they did not*)

Group	Group-generated criteria for dissolving ionic solids			
	Separation of salt into ionic species	Salt becomes aqueous	No chemical change occurring	Presence or absence of water from the chemical equation
1A	+	+	–	+
1B	+	+	–	–
1C	–	–	–	+
1D	+	–	+	+
2A	+	+	+	+
2B	+	+	–	+
2C	–	–	–	–
2D	+	–	+	+
3A	+	+	–	–
3B	–	–	–	+

Given that chemical justifications became normative in each group’s discourse and were used repeatedly by a large number of the groups as a guide to select the correct multiple-choice option, we examined the patterns of criteria used across the groups. The next section shows the results of this analysis.

### Across Group-Negotiated Criteria (AGC)

One criterion we used to determine whether an idea became normative was to examine if it was consistently used as a chemical justification by the ten different groups in the study (code AGC, Table 1). That is, we looked for similarities and differences in meaning-making across the groups. One criterion, the absence or presence of water from the representative chemical equation, focused more on the symbolic features of the chemical equation. The other three criteria described in Table 4 (separation of ionic salts into ionic species, becoming aqueous, and absence of chemical change) represent physical properties of dissolving ionic compounds. The following paragraphs describe patterns of criteria use across the groups.

### Patterns of criteria use—focus on symbolic features in equations

Seven of the ten groups in the study used the presence/absence of water as a criterion, often in combination with other criteria (Table 4). When groups focused on water, they were using it either as an exclusionary criterion (water did not change or the chemical equation does not show water) or as a confirmatory criterion (this chemical equation shows water and therefore must be the correct option). This point is illustrated in the sociochemical dialog of group 1C whose only criterion was water. A partial dialog from group 1C is shown in Table 5.

**Table 5:** Part of group 1C’s dialog on choosing the correct equation to represent the dissolution of sodium chloride (NaCl) in water

Dialog	Code	Comments/interpretations
1 SI: For one thing it’s the only one you’re adding water to. The other ones aren’t adding water to it. They are have an equation without water	GNC/AGC	Provides chemical justification why option be would be correct
2 S3: It’s definitely not D and E. I don’t think it’s A or C because they don’t have the water in the equation	GNC/AGC	Confirmatory Consensus-checking statement
3 Okay [group chooses B and moves on to the next activity]		Acknowledgement statement

In line 1 of Table 5, *S1* argues option B is the only correct option because “it’s the only one you’re adding water to. The other ones aren’t adding water to it. They have an equation without water”. In confirmatory, consensus-checking statement, *S3* uses the presence of water in the equation as a confirmatory evidence to rule out the other possibilities stating emphatically “It’s definitely not D and E. I don’t think it’s A or C because they don’t have the water in the equation” (LN 20). Similar arguments were made by students in other groups who used water as a confirmatory criterion. For instance, members *S2* and *S4* of group 1D focused on water as a confirmatory criterion to justify their response. Member *S2* stated “The only one that makes sense is B because it’s the only one that is adding water” while *S4* argued “It’s not D or E because those are both solids. It can’t be those two. B is the only one that makes sense because it’s the only one adding the water.” It is interesting that group 1C and group 1D used similar language to justify their responses on why option B is correct.

### Patterns of criteria use—focus on physical properties

Seven of the ten groups in this study used the idea that ionic salts separate into ions when dissolved in water as a criterion (Table 4). This was the most frequent reasoning groups used to justify their selection of which chemical equation best represented the dissolution process. Because students used a molecular model kit in which they simulated the dissolution process of ionic salts by using magnetized NaCl and water molecules which upon mixing dissociate (opposite magnets get attracted to each other), it is feasible the molecular model kit influenced their view. Groups that proposed separation of salts into ions were also likely to justify their responses by reasoning that salts become aqueous when added to water. Four of the five groups that used ‘aqueous’ as criterion similarly used separation of ionic solids into ions as criterion (see Table 4). These two criteria combined describe the physical properties of dissolving ionic solids.

### Developing criteria for chemical conductivity

In the preceding section, we described how students grappled with how to represent the dissolution of ionic solids in water at the symbolic level. The groups, however, also used chemical bonding models and information about overall molecular structure as a criterion for describing the properties of ionic compounds in solution. This was particularly evident in the groups’ sociochemical dialogs with respect to electrical conductivity. Table 6 shows an illustrative example in which group 1A discussed the conductivity of chemical compounds in water. The context for the episode was as follows: once students completed symbolic and particulate representations of dissolved ionic solids, they were prompted to predict the electrical conductivity of sodium iodide (NaI, an ionic compound) and sucrose (C_12_H_22_O_12_, a molecular compound) in water.

**Table 6:** Group 1A’s dialog on predicting conductivity of NaI and sucrose

Dialog	Code	Comments/interpretations
1 S2: The first one would definitely [conduct] because it’s ionic	GNC/AGC	Initiating move-justification based on molecular structure
2 S1: Because the molecule is ionic? Or how do we explain it?	GNC/AGC	Seeks group consensus
3 S2: Does it say explain it or just predict?	Procedural	Procedural talk
4 S3: Predict with an explanation	GNC/AGC	Response statement
5 S4: The charge rule for that one.	GNC/AGC	Proposes ways to justify response
6 S2: When freely moving ions are present	GNC/AGC	Use of particular-level information
7 S1: I need to write it on the sheet, so how do you guys want to phrase it?	GNC/AGC	Seeks group consensus
S4: I'd put that it will conduct electricity in an aqueous solution because it’s an ionic bond so the Nal compound would change to Na+ ions and I- ions and the particles would be surrounded by water, which conducts electricity	GNC/AGC	Follow-up statement: provides justification based on molecular structure information as a reason for response
9 S1: So. when added to water it will change to ions?	GNC/AGC/SST	Skeptical questioning-could signal change in thinking
10 S4: Yeah. For the second one do we think it will conduct or won’t conduct?	GNC/AGC	Asks for group clarification
11 S2: I don’ think it would because it’s a molecular compound	GNC/AGC	Provides structure-based argument for why suggestions is correct
12 S3: It will stay together	GNC/AGC	More structure-based evidence
13 S4: Yeah		Acknowledgement /support
14 S1: So. the second one will not conduct electricity?	GNC/AGC	Group consensus-checking statement
15 S2: I kind of did an experiment like this in high school and it was like this	Side Talk	brings in previous experience
16 S1: Because it’s a molecular compound and won't separate?	GNC/AGC	Group consensus-checking statement
17 S4: Yeah [Whole class interruption by course instructor]	GNC/AGC	Acknowledgement statement
18 S4: I was saying it was a molecular compound and it won’t separate into ions when water is added. It will stay a compound	GNC/AGC	Response statement; affirms group response
S1: That sounds good	GNC	Acknowledgement statement

In the initiating statement of the episode, *S2* stated “the first one [NaI] would definitely [conduct] because it is ionic” (LN 1). *S2* justified her reasoning of why NaI would conduct electricity based on molecular structure information (“because it is ionic”). *S1* who was the group’s recorder asked for clarification: “because the molecule is ionic?” (LN 2). *S1* refers to NaI as “the molecule” and asked if “the molecule” being ionic was sufficient to explain why NaI would conduct—“how do we explain it?” (LN 2). Clearly, in addition to developing criteria that would help them determine which molecules will conduct and which ones will not, it is evident from the group’s dialog that they were involved in group thinking.

In line 5 of Table 6, *S4* suggested using “the charge rule” as an explanation for why NaI would conduct electricity. Here, *S4* referred to the presence of positive charges in sodium ions and negative charges in iodide as the “charge rule.” This was further described by *S2* who stated in line 6 “when freely moving ions are present…” although she was interrupted before she could finish her thought. *S4*’s suggestion of “to use the charge rule” appears to have prompted *S2*’s response in line 6. *S1* asked for a group consensus: “I need to write it on the sheet, so how do you guys want to phrase it?” (LN 7). *S4* responded by saying “I’d put that it [NaI] will conduct electricity in an aqueous solution because it’s an ionic bond so the NaI compound would change to Na+ ions and I- ions and the particles would be surrounded by water, which [water?] conducts electricity” (LN 8). Clearly, *S4* had a good grasp of conductivity, as evidenced by her reference to NaI dissociating into charged particles which when surrounded by water will conduct electricity. In line 9, *S1* asked skeptically “So, when added to water it will change to ions?” *S4* responded by saying “Yeah” (LN 10).

In the exchange in lines 1–10, all members of the group participated in the ongoing dialog (although *S3*’s contribution is limited to one line (LN 12), it is important and led to extended discourse). With respect to NaI, there are two ideas which the group comes back to: (1) there is an ionic bond in NaI and (2) there will be freely moving ions when the ionic NaI is in an aqueous environment. The group uses both of these ideas as criteria to justify their response with respect to the conductivity of NaI.

The group’s discourse with respect to sucrose was initiated by *S4* in line 10 of Table 6 who asked “for the second one [sucrose] do we think it will conduct or won’t conduct?” *S2* responds by saying “I don’t think it would” (LN 11) and provided structure-based justification for her reasoning “because it’s a molecular compound” (LN 11). *S3* further elaborated on why sucrose will not conduct electricity by stating—“it [sucrose] will stay together” (LN 12). *S4* acknowledged the conclusions her colleagues reached by simply stating “Yeah” (LN 13). Here, we see the group provide justifications for their responses based on molecular structure information (i.e., compound stays together; it’s a molecular compound). In lines 10–13, there was no mention of “freely floating ions” or “the charge rule”—the ideas the group associated with sodium iodide.

The group’s repetitive use and appeal to molecular structure suggests structure-based reasoning became normative within the group’s dialog and they used structure-based reasoning to justify their explanation of why NaI would conduct electricity while sucrose would not. In lines 14 and 16, *S1* checked for group consensus “so, the second one will not conduct electricity”—and the reason for that explanation “because it’s a molecular compound and won’t separate?” *S4* confirmed this conclusion when she states “yeah” (LN 17). *S1*’s consensus-checking statements (LN 9, LN 16) reframed the group’s responses in terms of structure-level descriptions (“when added to water it will change into ions?”, “it’s a molecular compound”).

### Extension to the remaining groups

We analyzed the data for the remaining groups in a manner similar to the procedure described above for group 1A. Table 7 summarizes the most frequent justification used by the groups to account for the conductivity of NaI and sucrose. The results of this analysis provided further evidence that the groups used structure-based justification to predict whether sodium iodide and sucrose will conduct electricity.

**Table 7:** Summary of groups’ most frequently used justifications for predicting NaI and sucrose conductivities in water

Group #	Group’s prediction and reasoning of NaI conductivity in water		Group’s prediction and reasoning of sucrose conductivity in water	
	Group’s prediction and reasoning of NaI conductivity in water			
	Prediction	Reasoning	Prediction	Reasoning
1A	Conducts	• NaI is ionic compound that will separate into ions	Would not conduct	• It is a molecular compound and will stay together
1B	Conducts	• NaI is ionic compound that will separate into ions	Would not conduct	• It is a molecular compound and will stay together
1C	Conducts	• NaI is ionic compound that will separate into ions in water	Would conduct	• Water molecules will still be touching and water conducts
1D	Conducts	• NaI is ionic compound that will separate into ions	Would not conduct	• It is a molecular compound and will stay together
2A	Conducts	• NaI is ionic compound that will separate into ions	Would not conduct	• It is a molecular compound and will stay together
2B	Conducts	• NaI is ionic compound that will separate into ions	Would not conduct	• It is a molecular compound and will stay together
2C	Conducts	• Salt water conducts	Would conduct	• Water molecules will still be touching and water conducts
2D	Conducts	• NaI is ionic compound that will separate into ions	Would not conduct	–
3A	Conducts	• NaI is ionic compound that will separate into ions	Would not conduct	• They are all non-metals
				• There is not a metal bond between the ions
3B	Conducts	• NaI will dissolve completely	Would not conduct	• If you double the molarity, you double [conductivity] values

As can be seen in Table 7, all groups predicted NaI would conduct. Most (7/10) initially predicted sucrose would not—groups 1C, 2C, and 2D were the exceptions and initially predicted either sucrose will conduct (1C and 2C) or made no prediction (2D). Overall, groups used similar language to describe the conductivity of both NaI and sucrose. With respect to NaI, they often used the terms “ionic compound” and different variation of “dissociation into ions” across the board as the reason why this compound would conduct electricity. The groups appeared to provide more details in justifying the reasons for their prediction of why sucrose would not conduct electricity. Their justifications ranged from ‘it is a molecular compound’ to ‘there are no free moving ions.’ Their responses with respect to sucrose appeared to be more nuanced than their discussion on why NaI would conduct. Nevertheless, both responses indicated routine uses of structure-based justifications in their predictions of electrical conductivity.

## Discussion

It is apparent from the groups’ sociochemical dialogs that students were involved in chemical meaning-making and negotiated criteria to support their understanding of the dissolution of ionic and molecular compounds in water. Our first research question explored the development of sociochemical norms that regulate classroom discourse on what counts as an acceptable chemical justification. Individual groups repeatedly used the idea of ionic solids in water separating into ions and becoming aqueous as justification for their selection of an appropriate symbolic equation to represent the dissolution of NaCl in water. Another chemical criteria developed by groups was that dissolved ionic compounds do not undergo a chemical transformation upon dissolving. The recurrent use of these chemical ideas (ions, aqueous, chemical change) provided evidence for the presence of sociochemical norms, a normative type of reasoning based on chemical justifications evident in group discourses. This finding particularly highlights how inquiry-based materials (i.e., POGIL) enabled autonomous student groups to develop sociochemical norms related to the dissolution of ionic solids.

In addition to developing criteria based on the physical properties of dissolving ionic solids (i.e., ions, aqueous), student groups also developed criteria focused on the symbolic presence of water in chemical equations representing the dissolution process. The ways in which groups focused on water in their dialogs suggest students relied on algorithmic understanding to represent the dissolution of ionic solids in water without understanding the underlying chemical principles—e.g., the application of double displacement reactions in the wrong context. This finding is consistent with previous literature highlighting students’ inability to facilely shift between symbolic and particulate representations of chemistry (Johnstone 1991; Naah and Sanger 2013). The questions in the POGIL activity used in this study elicited discourse related to the focus on water in the symbolic equations and subsequently led to shifts in student understanding. This study, therefore, highlights the need to engage students in instructional activities that not only engage them in the multiple ways of representing chemical knowledge but also making public their views and participating in discourse.

The benefits of collaborative discourse revealed in this study parallel the findings of previous research (Osborne 2010). However, research on how group thinking influences individual student learning is rare. Thus, our second research question examined the influences of classroom discourse on individual student’s understandings of ionic compounds in solution. Individual students in this study shifted their thinking following group discourse. Many students were insistent that the symbolic equations representing the dissolution of ionic solids needed to show water molecules. As described above, we suspect these students were relying on algorithmic understanding of chemical reactions without connecting to the particulate level. This data suggests that individual students working on their own are not likely to come to the same representations of ionic solids at the symbolic and the particulate level that a cooperative learning group would. The unfolding sociochemical dialogs in the groups, including confirmatory and challenging statements made by individual members in a given group, led to shifts in student understanding.

This study showed that students were able to collectively develop criteria on what counts as acceptable and justifiable reasoning for selecting appropriate symbolic equations for dissolving ionic solids and what accounts for the conductivity of chemical materials in water. This collective development of ideas highlighted the influences of social factors and collaborative discourses on student learning. We suggest students’ verbalization of their ideas provided opportunities for constructive discourse and enhanced their conceptions of the dissolution of ionic solids in water. Thus, activities that facilitate students to make public their views and debate opposing views during unfolding sociochemical dialogs avail students opportunities to shift their thinking. Group dialogs showed the evolution of individual thinking when students were confronted with opposing viewpoints with respect to the nature of dissolving ionic solids in water.

An alternative explanation to the idea that students in this study are developing chemical justification of what happens to ionic solids placed in water is that they may be simply articulating what the products are in the multiple-choice question choice question (i.e., one way to verbalize the answer choice Na + (aq) and Cl−(aq) is that they separate into ions and become aqueous. That is, the students are using simply using language consistent with the chemical representation in the correct answer choice—i.e., they are verbally interpreting the symbolic answer in a multiple-choice question. We suggest analysis of pre-activity assessment a day before the POGIL activity argues against this plausibility. In the immediate pre-assessment (see Additional file 1: Figure S1), analysis suggested the majority of students (55%) held the view that dissolving ionic solids chemically react with water to form an acid and a metal oxide or hydroxide (Additional file 1: Figure S1, option C), a common chemical misconception reported in the chemistry education literature (see Naah and Sanger 2013). The second most popular option in the pre-assessment data suggests students thought ionic solids combine with water to form one large molecule (21% chose option E in Additional file 1: Figure S1). Only 15% of the students were choosing the correct option that shows ionic solids separate into ions when dissolved in water (option A, Additional file 1: Figure S1) in the pre-assessment data. This suggests that the sociochemical dialogs within the POGIL ChemActivity influenced students’ understanding. That is, the collective development of understanding, the group’s chemical meaning-making in social context, and the use of structure-based justification to explain physical and chemical properties availed individual students opportunities to shift their thinking and challenge and provide immediate feedback to members of their social group.

### Symbolic interactionism and meaning-making processes

In the foregoing discussion, we described how activities that promoted group reasoning about chemical ideas led to the development of sociochemical norms that regulated classroom discourse and the nature of student engagement. In keeping with the symbolic interactionist perspective (Blumer 1969; Yackel and Cobb 1996), we note how every situation was negotiated anew through student interactions during small and whole-class discussions. In this sense, interpretations of everyday words such as “breaking up” took different meaning as the participants interacted with each other. These “re-interpretations” led to the development of specific criteria that guided the groups’ reasoning about the nature of dissolved ionic compounds. However, as Yackel and Cobb (1996) notes, to the extent norms taken-as-shared were negotiated within and across the groups in this study, it was evident there was stability in the new reinterpretations of words and ideas such as “breaking up” across and within the groups. This suggests that there are normative aspects of classroom environment that chemistry instructors can expect when teaching in cooperative and inquiry settings.

## Study limitations

While this study showed influences of group dialogs on individual student learning, there are two limitations that are worth noting. The first limitation is of methodological nature, our analysis relied on recordings of student discussions that allow us to only access what students verbalize, which is not necessarily identical to what they think. Yet, verbal cues are the major mechanism we use to communicate. In that sense, while the method of relying on recorded discussions allows us only hear what students verbalize, we can never know what somebody thinks without trusting the words used to communicate that thinking. The second major limitation of the study is the fact that this data comes from a single university and institution and a particular classroom within that context. Thus, results of the study cannot be generalized to other settings without replication of similar analysis in diverse settings and institutions.

## Conclusion

Findings on the development of sociochemical norms and the ways in which chemical ideas were used in the cooperative learning groups in this study have implications for science teaching. Understanding the how’s and why’s of student learning can help science educators understand the dynamics of and the social factors that influence the classroom learning environment. Wu (2003) suggests that authentic features of a curriculum need to emphasize the establishment of social norms (and in this case sociochemical norms) that shape and influence student learning. In setting up scenarios that generate sociochemical dialogs, it is important to consider the structure of inquiry materials and how they elicit student conceptions of chemical ideas and understandings. This suggests paying attention to the nature of the prompts in inquiry materials and how they elicit group dialog—e.g., writing prompt questions that provide contradicting explanations such as questions that rely on only algorithmic understanding vs. particulate explanations. The study also highlights the importance of classroom discourse and the role social factors play in shaping student understanding of chemical ideas. This suggests the importance of establishing classroom environments that support group dialog and social interactions, such as the POGIL classroom in which this study was conducted.

This study provides a backdrop for further research to explore patterns of student reasoning in the context of sociochemical dialogs and how instructional materials can improve student understanding of chemistry. An important question worth asking is: Do the observed sociochemical norms lead to shifts in individual student conceptions of ionic compounds in solution? We plan to address this question in a future paper that examines if the groups’ sociochemical dialogs resulted shifts in student understanding of dissolution processes based on the observation that when students are confronted with opposing viewpoints with respect to either dissolution process or electoral conductivity, they seem to change their initial thinking. Other questions worth pursuing include: What promotes effective sociochemical dialogs that lead to shifts in student understanding? Are there specific patterns to curricular materials that promote effective student discourse? Another observation in this study was the way the different autonomous groups used the criteria they developed to describe the dissolution process of ionic compounds. For instance, some focused on the symbolic features present in the equation while others used abstract chemical ideas. Therefore, it will be worthwhile examining what discourse patterns in student-initiated sociochemical dialogs promote or constrain student development of criteria to explain given chemical phenomena. The methodology developed by Cole et al. (2012) based on Toulman argumentation model (Toulman 1958) is one way to approach addressing these questions. The nature of the analysis done in this work, which does not utilize Toulman argumentation, provides an alternative methodological approach to investigate these questions and may provide insights into how classroom discourse in which students interact with each other influence students’ learning of chemical ideas.

## Additional file

Additional file 1: **Figure S1.** Sample pre-assessment data and student responses on what happens to ionic solids when placed in water. The pre-assessment data was collected the day before the POGIL activity and suggests most students held common misconceptions about the nature of what happens to ionic solids dissolved in water. (TIF 1408 kb)

## Abbreviations

AGC: Across Group-Negotiated Criteria
GNC: Group-Negotiated Criteria
POGIL: Process oriented guided inquiry learning
STEM: Science, Technology, Engineering, and Mathematics
