# Supplementary figures and images for: The influences of group dialog on individual student understanding of science concepts
Source: Int J STEM Educ. 2018 Nov 3;5(1):46. doi: 10.1186/s40594-018-0142-3 (PMC6310434; doi:10.1186/s40594-018-0142-3)

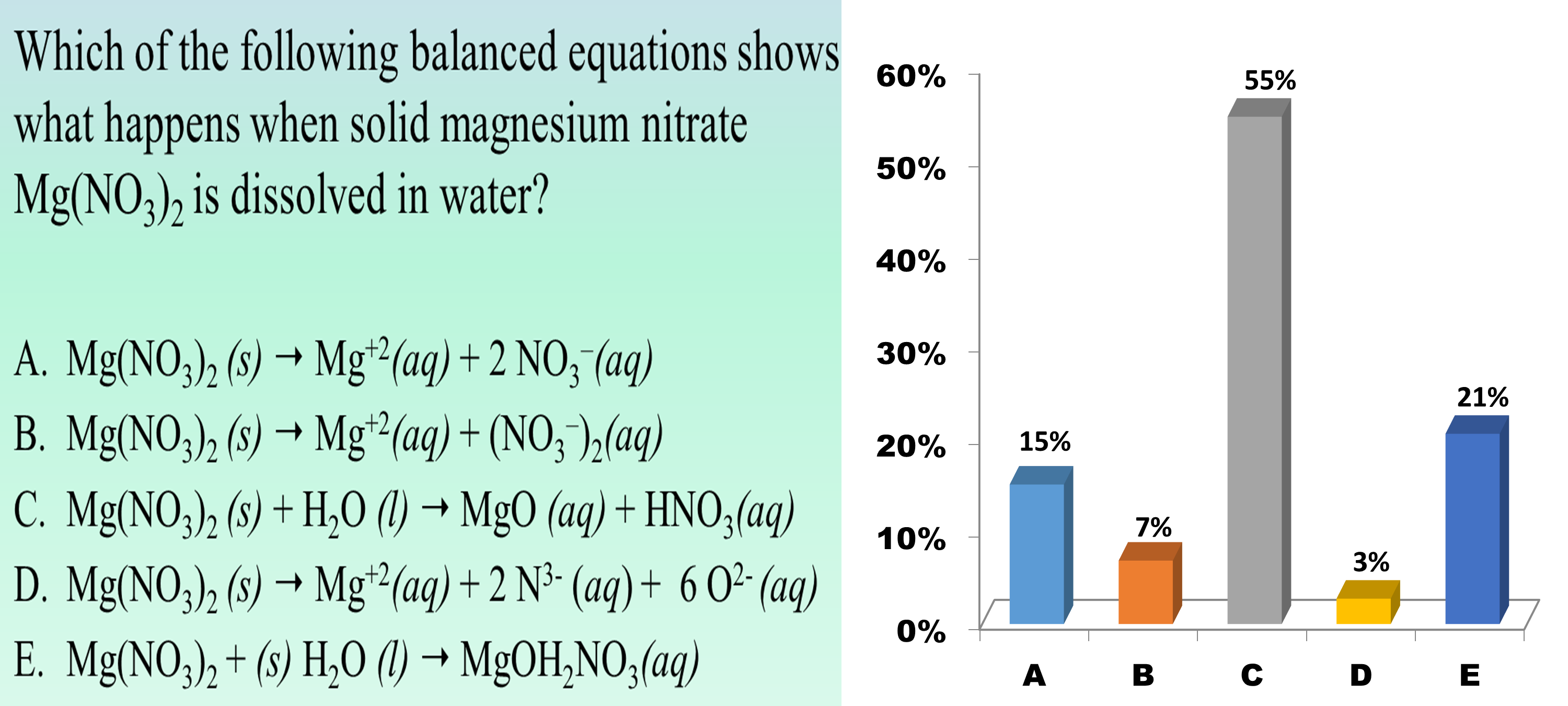

Supplement: Supplementary file 1 — Figure S1. Sample pre-assessment data and student responses on what happens to ionic solids when placed in water. The pre-assessment data was collected the day before the POGIL activity and suggests most students held common misconceptions about the nature of what happens to ionic solids dissolved in water. (TIF 1408 kb) [file 40594_2018_142_MOESM1_ESM.tif]
